# Development and Acceptability of a Tablet-Based App to Support Men to Link to HIV Care: Mixed Methods Approach

**DOI:** 10.2196/17549

**Published:** 2020-11-24

**Authors:** Thulile Mathenjwa, Oluwafemi Adeagbo, Thembelihle Zuma, Keabetswe Dikgale, Anya Zeitlin, Philippa Matthews, Janet Seeley, Sally Wyke, Frank Tanser, Maryam Shahmanesh, Ann Blandford

**Affiliations:** 1 Africa Health Research Institute KwaZulu Natal Mtubatuba South Africa; 2 Department of Sociology University of Johannesburg Johannesburg South Africa; 3 Department of Health Promotion, Education and Behavior University of South Carolina Columbia, SC United States; 4 UCL Interaction Centre University College London London United Kingdom; 5 London School of Hygiene and Tropical Medicine London United Kingdom; 6 University of Glasgow Glasgow United Kingdom; 7 Lincoln International Institute for Rural Health University of Lincoln Lincoln United Kingdom; 8 Institute for Global Health University College London London United Kingdom

**Keywords:** HIV, linkage to HIV care, digital technologies, men, mobile phone

## Abstract

**Background:**

The poor engagement of men with HIV care is attributed to a number of factors: fear of stigma, masculine representations, concerns related to confidentiality, and the time commitment needed to visit public health clinics. Digital technologies are emerging as an approach to support the engagement of men with care.

**Objective:**

This study aims to deliver a usable and engaging tablet-based app, called EPIC-HIV 2 (Empowering People through Informed Choices for HIV 2), to support men in making informed decisions about engaging with HIV care in rural KwaZulu Natal, South Africa.

**Methods:**

We employed a mixed methods, iterative, and three-phased design that was guided by self-determination theory (SDT), a person-based approach, and human-computer interaction techniques. We reviewed related literature and conducted secondary analyses of existing data to identify barriers and facilitators to linkage to care and inform content development and design principles and used focus group discussions with members of the community advisory board and general community to evaluate a PowerPoint prototype of the app; used observations and guided questions with a convenience sample of potential users from the intervention community to iteratively test and refine a functioning interactive app; and conducted qualitative interviews and satisfaction surveys with actual users to evaluate acceptability.

**Results:**

Phase 1 identified supply- and demand-side barriers to linkage to care. Specifically, clinics were feminized spaces unattractive to men with high social costs of attendance. Men did not feel vulnerable to HIV, preferred traditional medicine, and were afraid of the consequences of being HIV positive. Thus, the app needed to allow men to identify the long-term health benefits to themselves and their families of starting antiretroviral therapy early and remaining on it, and these benefits typically outweigh the social costs of attending and being seen at a clinic. SDT led to content design that emphasized long-term benefits but at the same time supported the need for autonomy, competence, and relatedness and informed decision making. Phase 2 indicated that we needed to use simpler text and more images to help users understand and navigate the app. Phase 3 indicated that the app was acceptable and likely to encourage men to link to care.

**Conclusions:**

We found that iteratively developing the app with potential users using local narratives ensured that EPIC-HIV 2 is usable, engaging, and acceptable. Although the app encouraged men to link to HIV care, it was insufficient as a *stand-alone intervention* for men in our sample to exercise their full autonomy to link to HIV care without other factors such as it being convenient to initiate treatment, individual experiences of HIV, and support. Combining tailored digital interventions with other interventions to address a range of barriers to HIV care, especially supply-side barriers, should be considered in the future to close the present *linkage gap* in the HIV treatment cascade.

## Introduction

### Poor Engagement of Men With HIV Care

Poor engagement of men with HIV care is well described [1-5]. This results in the persistence of unacceptably high HIV-related mortality among men and new HIV cases in young women [6]. In rural KwaZulu-Natal, South Africa, despite freely available antiretrovirals (ARVs) in the public sector, men are 25% more likely than women to die of HIV-related illnesses, and 70% of them had never sought care [7]. Fear of stigma, cultural constructions of masculinity that do not involve caring for health, concerns related to confidentiality, and time commitment needed to attend public health clinics have been cited as key reasons for men’s reluctance to get tested and start HIV treatment [1,8]. Novel, scalable interventions that improve the uptake of HIV testing and care among men are needed to improve individual health and reduce HIV transmission risk [9,10].

### The Potential of Digital Technologies in Closing Gaps in HIV Care

Digital technologies such as mobile phones are emerging as one of the approaches to close the gaps across the HIV care cascade [11,12], especially in light of the expanding access to smartphones in areas most affected by HIV/AIDS. A recent Pew internet survey found that 33% of respondents from sub-Saharan Africa owned a smartphone, and the number is expected to double by 2025 [13]. Digital technologies are particularly suitable as interventions to help prevent and manage HIV and AIDS, as they have the potential to access and impact hard-to-reach populations, including those who typically feel stigmatized within health care settings [14]. Daher et al [15] conducted a systematic review of digital innovations for HIV or sexually transmitted infections and found them to be effective in improving clinic attendance, adherence to antiretroviral therapy (ART), and turnaround time from testing to treatment. This suggests that digital interventions have considerable potential to support engagement in the HIV care continuum.

### Challenges to the Development of Digital Interventions

There are many challenges to the development of digital interventions: most importantly, ensuring that the digital platform is usable and engaging for end users [16] and delivering the intended messages. Designing digital interventions for resource-constrained settings such as rural South Africa has added challenges, as there are varying levels of education, health, and digital literacy [17]. Furthermore, ensuring that the intervention supports individual decision making can be challenging. There are limited data on how to develop digital interventions for HIV care in resource-constrained settings that are usable and engaging and support informed individual decision making.

As part of a larger, cluster randomized controlled clinical trial to increase home-based HIV testing and linkage to care in men [18], we developed 2 interactive tablet-based apps (to integrate with the trial) called EPIC-HIV 1 and 2 (Empowering People through Informed choices for HIV 1 and 2). Both are guided by self-determination theory (SDT) to support informed decision making and make explicit and usually implicit processes of decision making. EPIC-HIV 1 is offered at the point of HIV test to help men make an informed choice about testing and if necessary linkage to care. EPIC-HIV 2 is offered to men who have tested HIV positive but have not linked to care within a specified time frame. It aims to encourage men to link to care and stay in care by supporting autonomy, feelings of relatedness, and competence in linking to care by using positive examples from other men living with HIV.

### Theoretical Framework

The primary aim of EPIC-HIV 2 is to support informed decision making by making explicit decisions that are usually implicit and thus, encourage men to link to care, to initiate ART early, and to introduce the potential benefits of staying on ART. Therefore, it is aimed at shifting the motivation of link to care from unmotivated or a motivated to internally motivated because of the benefits of ART. SDT addresses factors that either facilitate or undermine motivation. According to SDT, human motivation is based on the satisfaction of 3 inherent psychological needs: (1) autonomy, (2) competence, and (3) relatedness. Autonomy requires an individual to make personal decisions and act in a way that corresponds to their identity, belief systems, and values [19,20]; competence refers to an individual’s perceived ability to perform an act [21]; and relatedness refers to the ability of an individual to connect to others and feel cared for [22]. SDT proposes that events or conditions that enhance a person’s sense of autonomy, competence, and relatedness support internal motivation, which, in turn, can facilitate the adoption of new behaviors to be internally motivated and sustained [20,21].

In the case of HIV, SDT has been used to understand ART adherence, treatment motivation [23], and overall health behavior among individuals living with HIV [22,24]. Applying evidence-based theories to the development process of interventions helps to direct attention to design characteristics that may otherwise be ignored and indicate conditions under which interventions are more likely to be effective [25]. EPIC-HIV 2 development was guided by SDT to increase men’s internal motivation to engage with HIV care. In this paper, we provide an overview of the approach we adopted for designing and developing EPIC-HIV 2. Using one component of the app as a detailed case study, we describe the design rationale and components of the app and report on its acceptability to users.

## Methods

### Study Context

As mentioned earlier, EPIC-HIV is being evaluated in a home-based intervention to test and start (HITS), a cluster randomized controlled trial designed to evaluate the effect of 2 interventions—EPIC-HIV (male-targeted HIV-specific decision support app) and microincentives on increasing home-based HIV testing and linkage to care among men, with the ultimate aim of reducing HIV-related mortality in men and hence HIV incidence in young women in rural South Africa [18]. The details of the study are described elsewhere [18] and is registered on the National Institute of Health trials (identifier NCT03757104). In summary, the trial uses the Africa Health Research Institute (AHRI) HIV surveillance platform, which visits households annually to conduct surveys using Android-based tablets and offer home-based HIV testing. EPIC-HIV 1 was administered at the point of HIV test offer during the fieldworker surveillance activity to encourage men to test for HIV. If a participant was diagnosed with HIV but did not present in the local Department of Health clinic within a month of the positive HIV test, a study fieldworker revisited the participant at home to offer EPIC-HIV 2. Both apps were installed on the field worker’s tablet. During the home visit, the field worker handed over the tablet to the participant together with earphones to allow the participant to independently explore the app. EPIC-HIV 2 uses a mixture of audio, text, video, still photos, and graphics and has different pathways through 3 interactive modules. This paper focuses on the design and development of EPIC-HIV 2.

### Study Design

**Figure 1:**
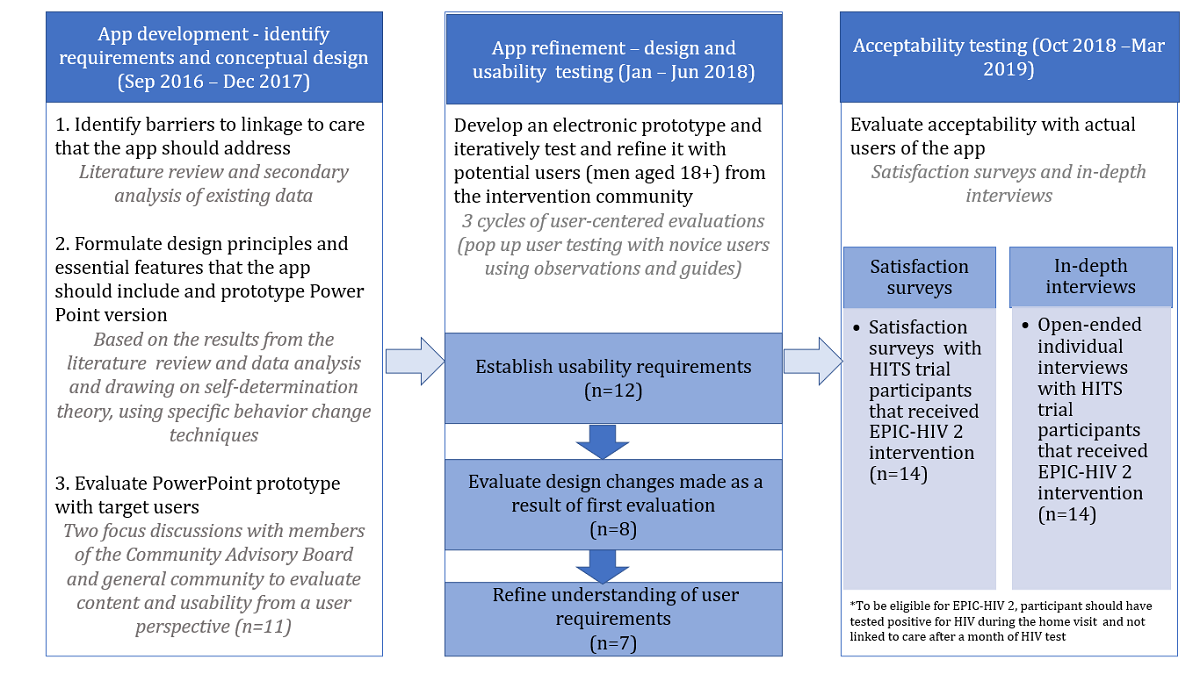
Mixed methods approach showing the 3 phases of the study. EPIC-HIV: Empowering people through informed choices for HIV; HITS: home-based intervention to test and start.

This was a mixed methods study using a person-centered approach and human-computer interaction (HCI) techniques. The study was conducted in 3 phases. Phase 1 focused on user requirements and early prototyping. It included identifying barriers and facilitators to HIV linkage to care through literature review and secondary analysis of existing qualitative data from AHRI. We then developed a PowerPoint prototype of the app based on SDT and drawing on established behavior change techniques [26], which was discussed with members of the Community Advisory Board (CAB; special body representing members of the intervention community that act as a bridge between AHRI and the community, safeguarding the rights of the study participants) and general members of the community in focus group discussions (FGDs). Together, these methods were used to inform app content requirements and conceptual design. In phase 2, based on the findings from phase 1, we developed a functioning, interactive app. We iteratively tested and refined the app with potential users from the intervention community using observations and guided surveys. Finally, in phase 3, we evaluated the acceptability of the app with actual users (participants who received the EPIC-HIV intervention in the HITS trial). This process is illustrated in Figure 1.

### Study Setting

This study was conducted in uMkhanyakude district in KwaZulu-Natal, South Africa. The area is predominantly rural and poor, with high levels of unemployment [27] and HIV prevalence [28]. Previous research has shown poor linkage and low ART uptake among men in this area [29,30] mainly because of fear of stigma [1,5].

### Recruitment and Sample

All participants were recruited from the AHRI HIV surveillance community. Each phase employed different recruitment and sampling methods; these are described in detail under each phase.

### Ethical Approval

Ethical approval for the study was granted by the Biomedical Research Ethics Committee of the University of KwaZulu-Natal (BREC 398/16).

Below, we provide an overview of each phase, outlining its main aim, study procedures, and participants.

### Phase 1: Requirements and Conceptual Design (App Development, September 2016 to December 2017)

#### Aim

The aim is to (1) gather the app content requirements and formulate design principles and features that the app should include by identifying modifiable barriers to linkage to care, (2) develop an initial prototype, and (3) evaluate it with potential users for overall impressions, appropriateness, and relevance to our setting.

#### Study Procedures

First, we drew on systematic reviews conducted on barriers and facilitators to testing and linkage to care and searched for additional studies in South Africa or elsewhere in sub-Saharan Africa (Multimedia Appendix 1 [1,31-43]). Second, we conducted a thematic secondary analysis from an AHRI study that explored community experiences and perceptions of tuberculosis and HIV transmission, treatment, and prevention [44] (Multimedia Appendix 1). The thematic coding frame also identified supply- and demand-side barriers to linkage to care, focusing on men’s perspectives. The findings from the literature review and secondary analysis were summarized as barriers to linkage, and those that were potentially modifiable were identified (Multimedia Appendix 1). Third, we identified specific behavior change techniques from the Behavior Change Taxonomy [26] that could support feelings of autonomy, relatedness, and competence and thus were likely to shift motivations for linkage to care, support informed decision making, and make explicit processes that are usually implicit (such as avoiding the clinic). Finally, we developed a PowerPoint prototype of the app and evaluated it with focus groups using an FGD guide (semistructured questions, assessing the content and design and overall app impressions). Informed consent was obtained from all participants before beginning each focus group. FGDs were conducted in the AHRI offices in isiZulu (native language), audio recorded, transcribed, translated, summarized by TZ, and discussed with the development team (MS, PM, SW, TM, and TZ). They were analyzed using thematic analysis [45] to draw out key themes for design.

#### Participants

To obtain diverse views, we invited a purposive sample of men aged 18 years and above from the CAB and general community. We sought members representing periurban and rural communities in the surveillance area. In general, we aimed for men of different ages and occupying various positions in the community. For example, we recruited men from the taxi industry, church, liquor business, and formal and informal occupations, those who were unemployed, and those in the traditional health sector. The AHRI public engagement (PE) department assisted with the recruitment of participants. Through PE, the research team explained the study to the CAB, and the members who were willing to participate and provide informed consent were phoned for further engagement and to arrange a suitable time for the FGD. Of the 10 CAB members, 7 were included in the study. To recruit community members, a research assistant in the study approached men meeting the study inclusion criteria and explained the study, and those who were willing to participate provided their telephone numbers to be contacted to arrange a time for the FGD. Of 11 men who were approached, 4 participated in the study. We conducted 2 FGDs, one for the CAB and one for the community members.

### Phase 2: Iterative Design and Usability Evaluations (App Refinement, January to June 2018)

#### Aim

The main aim of this phase is to iteratively design and test the app to refine it to be usable and engaging in our setting.

#### Study Procedures

We conducted 3 cycles of usability evaluations to assess participants’ ability to successfully navigate the app, comprehend the educational content, and determine whether they found the app to be engaging and relevant. The first cycle was to establish usability requirements, the second cycle was used to evaluate the design changes made as a result of the first evaluation, and the last cycle was used to refine the understanding of the user requirements. User-centered evaluations were conducted with potential users using observations and guided surveys that consisted of a mix of open and closed questions to assess comprehension of the content and record usability issues. The questions were adjusted to suit the objectives of each cycle of evaluation with 2 questions that remained consistent to benchmark the design changes (Multimedia Appendix 2). Evaluations were conducted in isiZulu, and we recorded the audio and tablet screen during the session. Data files were translated and transcribed, summarized, and discussed with the HCI expert and technological partner after each evaluation cycle. On the basis of the findings, the app was tailored to ensure that participants understood the messages correctly and to improve the user experience.

#### Participants

We used pop-up user testing [46] to recruit a convenience sample of men aged 18 years and above from the intervention community. We sought participants who represented a broad spectrum of technological and educational literacy, involving at least six participants in each cycle to get quick feedback on the app. The team drove (in an AHRI-marked vehicle) to different locations in the area and asked men (novice users) to participate in this study. Participants gave verbal consent and were asked to confirm if they were aged 18 years and above; they were not required to disclose their HIV status or any personal information.

### Phase 3: Acceptability Testing (October 2018 to March 2019)

#### Aim

The aim of this phase is to evaluate the acceptability and perceived value of the resulting app with actual users (participants who received the EPIC-HIV intervention in the HITS trial).

#### Study Procedures

We conducted satisfaction surveys and individual in-depth interviews. An adapted Client Satisfaction Questionnaire (CSQ-8) [47] was used to assess satisfaction with the app (see Table 1 for the adapted questionnaire). The CSQ-8 has 8 items: quality of app, app met needs, kind of service received from the app, recommend app to a friend, amount of help received from the app, effectiveness of the app for dealing with the health problem, overall satisfaction, and willingness to use the app again. These items are assessed on a 4-point Likert scale of 1 to 4 with individually specified anchors, and 4 is consistently the positive assessment. For this study, we added 2 questions to assess the relevance of the app for this setting and user friendliness. The CSQ-8 has been used to evaluate technology-based intervention and has demonstrated high consistency [48]. The 10 items were administered on a REDCap [49] project by a fieldworker. Proportions were used to describe the acceptability. Statistical analyses were conducted using STATA software (version 15.1; StataCorp LLC). We complemented the satisfaction surveys with in-depth interviews with a purposive sample of participants who received EPIC-HIV 2. The interview questions explored why participants had not been linked to care after the HIV diagnosis and their views of EPIC-HIV 2 and the impact it had on them. Interviews were conducted at participants’ homes in isiZulu and lasted approximately an hour. The audio files were transcribed, translated, and analyzed thematically with themes guided by SDT. NVivo was used to code and manage the transcripts. Separate informed consent was obtained for participating in the satisfaction surveys and in-depth interviews.

**Table 1:** Empowering People through Informed Choices for HIV 2 satisfaction surveys.

Survey item		Response, n (%)
**How would you rate the quality of the EPIC-HIV^a^ app you just listened to?**		
	Excellent	13 (93)
	Good	1 (7)
	Fair	0 (0)
	Poor	0 (0)
**To what extent has the app helped in meeting your needs with regards to information about HIV treatment?**		
	All	12 (86)
	Most	0 (0)
	Only a few	1 (7)
	None	1 (7)
**Did you get the kind of HIV management information you wanted or expected?**		
	Yes, definitely	14 (100)
	Yes, generally	0 (0)
	No, not really	0 (0)
	No, definitely	0 (0)
**Did the information you just listened to appeal to your conscience to go for HIV treatment and take ARVs^b^ if you have not done so?**		
	Yes, definitely	13 (93)
	Yes, generally	1 (7)
	No, not really	0 (0)
	No, definitely	0 (0)
**Do you feel empowered to make informed choices regarding your health with the information from the app?**		
	Yes, definitely	12 (86)
	Yes, I think so	2 (14)
	No, I don't think so	0 (0)
	No, definitely not	0 (0)
**If a friend were in need of HIV treatment and management information, would you recommend the app to him or her?**		
	Yes, definitely	14 (100)
	Yes, I think so	0 (0)
	No, I don't think so	0 (0)
	No, definitely not	0 (0)
**How satisfied are you with the amount of HIV management information you received from the app?**		
	Very satisfied	12 (86)
	Mostly satisfied	2 (14)
	Mostly dissatisfied	0 (0)
	Quite dissatisfied	0 (0)
**How would you rate the simplicity and user-friendliness of the app?**		
	Excellent	11 (79)
	Good	2 (14)
	Fair	1 (7)
	Poor	0 (0)
**In general, how satisfied are you with the app?**		
	Very satisfied	14 (100)
	Mostly satisfied	0 (0)
	Mostly dissatisfied	0 (0)
	Quite dissatisfied	0 (0)
**If you were to seek information about HIV management again, would you consider EPIC-HIV app?**		
	Yes, definitely	14 (100)
	Yes, I think so	0 (0)
	No, I don't think so	0 (0)
	No, definitely not	0 (0)

^a^EPIC-HIV: Empowering People through Informed Choices for HIV 2.

^b^ARV: antiretroviral.

#### Participants

All men aged 15 years and above who received EPIC-HIV 2 between October 2018 and January 2019, as part of the intervention in the HITS trial, were asked to consent and complete the satisfaction surveys. For the individual in-depth interviews, we purposively selected 14 men from the 28 who had received EPIC-HIV 2 between April 2018 and January 2019 (which meant that they had tested positive for HIV but did not link to care within a month).

## Results

Each phase is presented separately with a particular focus on the iterative development. Textbox 1 shows the key takeaway findings from the 3 phases.

**Key takeaway findings for the different phases.**

Phase 1

- Identified key learning points to incorporate in the content to persuade men to attend clinic and start antiretroviral therapy
- Long-term health benefits of starting antiretroviral therapy early and remaining on it

Phase 2

- Men in our setting did not understand the seesaw metaphor
- The click worked better than swipe
- Two options were better than 3 options

Phase 3

- App was usable and acceptable in our setting
- It encouraged men to link to HIV care

### Phase 1: App Development

**Figure 2:**
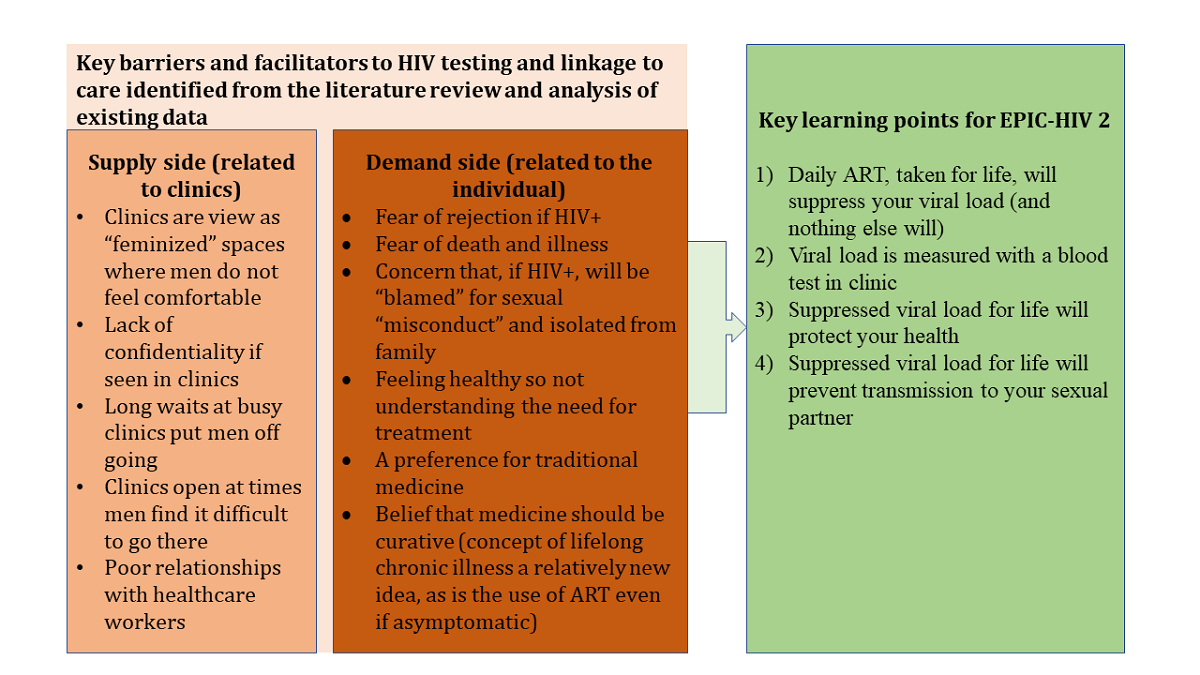
The identified key barriers and facilitators to HIV testing and linkage to care and the key learning points for EPIC-HIV 2. ART: antiretroviral; EPIC-HIV: Empowering people through informed choices for HIV; HITS: home-based intervention to test and start.

In the literature review and analyses of the earlier study, we identified barriers and facilitators to HIV testing and linkage to care among men, particularly relevant to our setting (Figure 2). Through this process, we identified 4 key learning points (shown in Figure 2) to be incorporated into the content of EPIC-HIV 2 with the aim of supporting informed decision making by making explicit decisions that are usually implicit and demonstrate that attending a clinic to start ART can be managed and that the long-term benefits of starting ART early and remaining on ART outweigh the costs. Drawing on SDT, we developed the app content and design to map to the psychological requirements for autonomy, relatedness, and competence (shown in Multimedia Appendix 3). To increase the likely personal identification with the messages and potential feelings of relatedness, the content was provided in the form of personal testimonies or experiences from local men. This was informed by evidence that suggests experiential information helps users make sense of what various outcomes might be like (in imagined futures) and increases their awareness of personal health risk and the likelihood of a response to the intervention [50].

A total of 11 men aged 34 to 66 years participated in 2 focus groups to evaluate the first (paper) prototype. They provided feedback on content and design (organization of the app, appropriateness and appeal of language and images, acceptability and relevance, and overall app impressions). On the basis of the feedback from the FGDs, the team modified the initial conceptual design of the app and created a revised plan for the app features and content.

**Figure 3:**
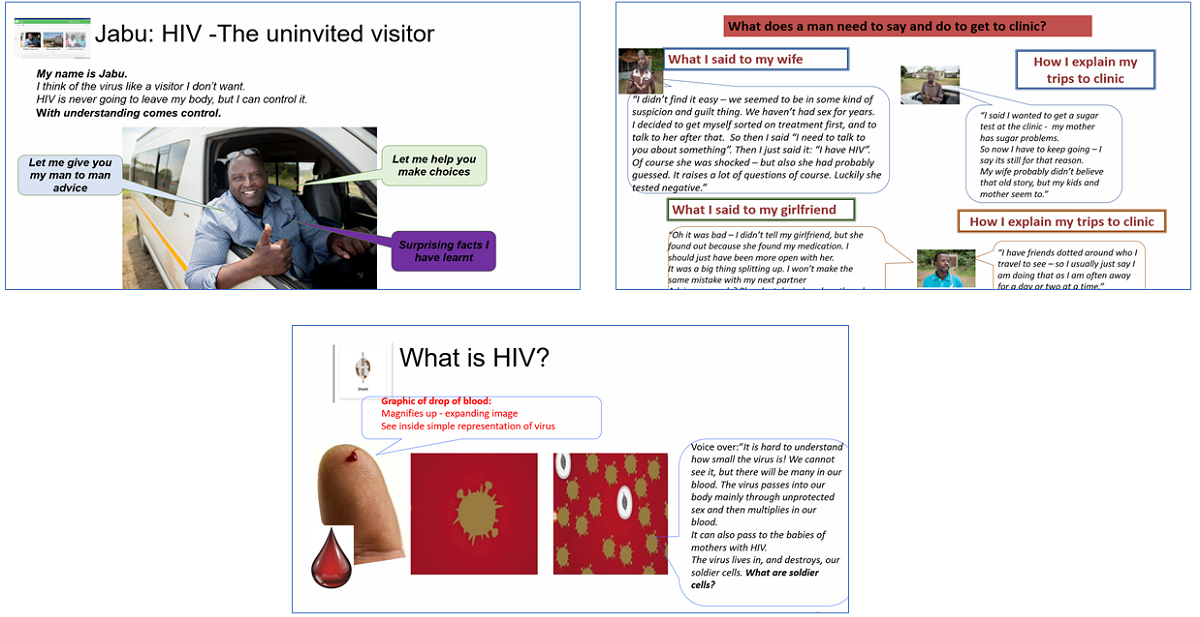
PowerPoint prototypes showing examples of the content from each module (presented in English, although all content was delivered in isiZulu in the final app).

**Figure 4:**
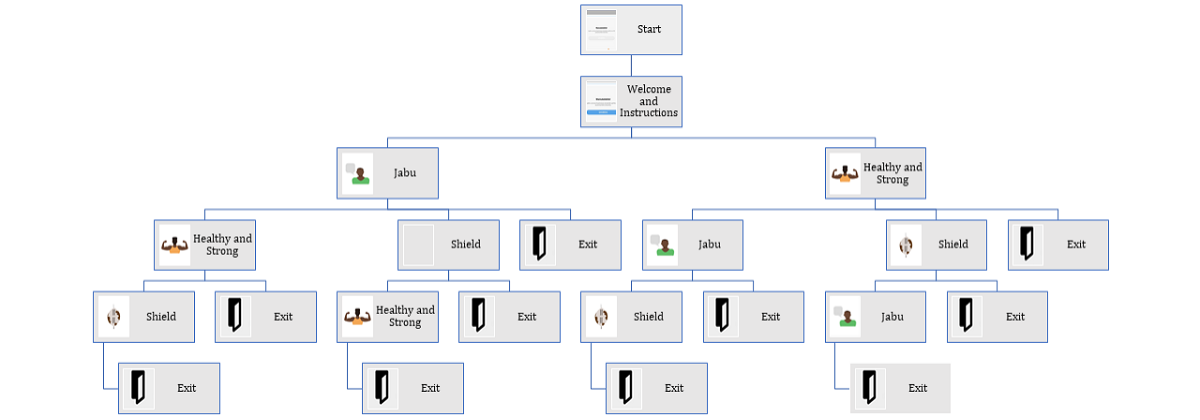
Map of the pathways through the 3 main modules of empowering people through informed choices for HIV 2.

The final realized product (EPIC-HIV 2) has 3 main modules (sections), and to further support the psychological requirements for autonomy, the user has the option to engage with 1 or all 3 modules (Figures 3 and 4; Multimedia Appendix 3). All 3 modules cover the 4 key learning points (Figure 2) but present the information in different ways to support different learning styles, social identities, and barriers to engaging with care: (1) Jabu’s man-to-man advice—a taxi driver talking about how he views HIV as an uninvited guest and gives his personal testimony on how he controls HIV; (2) healthy and strong with HIV—4 male characters who give their experiential information on how they managed going to the clinic, how they disclosed their HIV status, and how they started ART, and stayed on ART; and (3) the facts are your shield—gives a broader picture of ART and how it fights HIV in the body by explaining the commonly used medical terminology in HIV care. There is a start button, and a male voice over that introduces EPIC-HIV 2 and gives instructions for navigating the app. As mentioned earlier, the app uses a mixture of audio, text, video, still photos, and graphics, and there is a continue button that the user needs to click to move to the next page (Multimedia Appendix 4 provides an example of the navigation in the Jabu module).

The final conceptual design (realized in the product) starts with an introduction and instructions on how to use the app, and users are then able to choose between “Jabu’s man to man advice” or “healthy and strong with HIV” modules. The “shield” module was not offered initially because it has more focused material and is expected to appeal to fewer men (and to make the number of choices manageable). After users complete the initial module, they are returned to the menu and are able to choose one of the remaining modules or to end. Figure 4 shows all the possible pathways through the app. Each module was developed and tested individually, as described in phase 2, and once finalized, all the different modules were integrated into a single app that was evaluated for functionality and comprehension.

### Phase 2: Iterative Design and Usability Testing (January to June 2018)

#### Decision Support Tool

The main outcome of phase 1 was an in-depth understanding of user needs relevant to EPIC-HIV 2. We subsequently refined the prototypes using a standard app development toolkit to deliver testable interactive demonstrator prototypes on an Android tablet computer. In this paper, we illustrate the approach to iterative design and usability testing by focusing on the development of the decision support tool (a component within *Jabu*).

**Figure 5:**
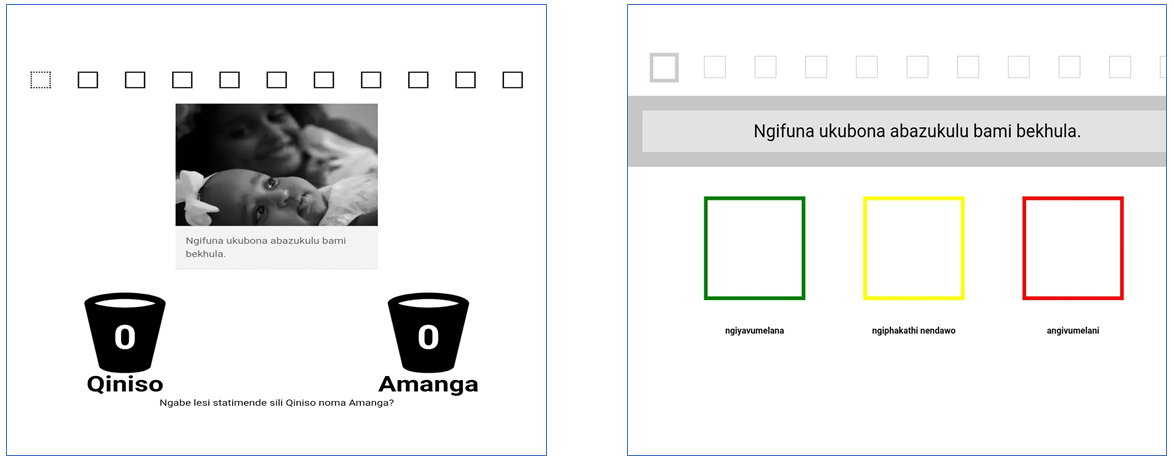
Example screenshots of drag-and-drop (statement with an image and illustration on how to drag) versus Likert scale (statement only).

**Figure 6:**
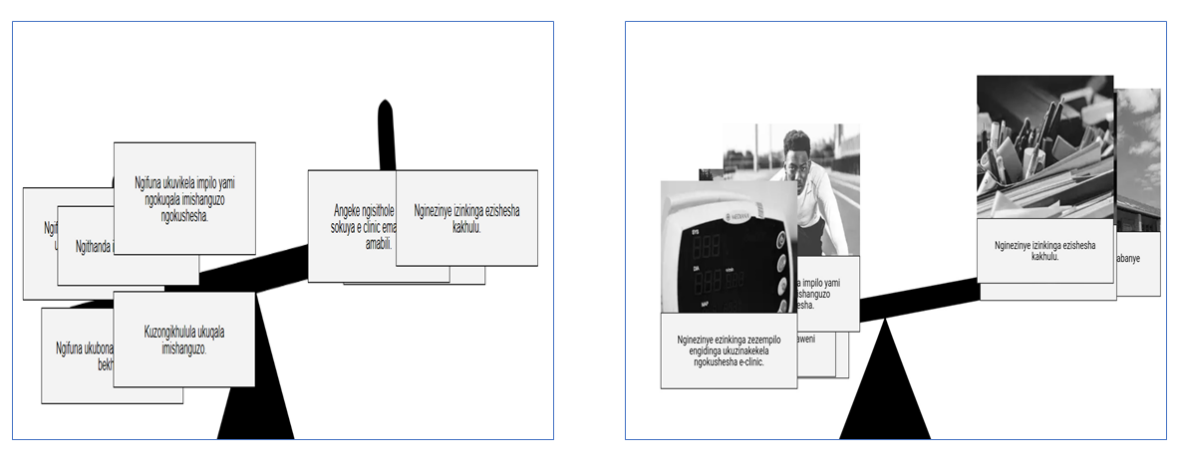
Example of the seesaw screenshots with statements only (from Likert scale version) versus statements with images (from drag-and-drop version).

The decision support tool aimed to help men weigh up and reflect on the costs and benefits of attending a clinic. The user was presented with different statements that either support or delay going to the clinic. To facilitate engagement, we wanted to make this section interactive. Initially, we applied a seesaw metaphor for users to attempt to render usually implicit decisions as explicit and interpret whether they were ready to attend a clinic within 2 weeks. This was done by adding the arguments to one side or the other of the seesaw to illustrate how the arguments stacked up in decision making. For example, if the user agrees that they want to see their children and grandchildren grow up, that would be an argument that weighs in favor of engaging with HIV care, whereas if they agree that they do not wish to be seen at a clinic by one of their neighbors, that would be an argument that weighs against engaging with care. In making design decisions about the clinic decision support tool, we prototyped and tested 2 versions: drag-and-drop with illustrations and a Likert scale without illustrations, both employing the seesaw metaphor. These are illustrated in Figure 5 (loose translation: “ngifuna ukubona abazukulu bami”—I want to see my grandchildren; “qiniso”—true; “amanga”—false; “ngiyavumelana”—I agree; “ngiphakathi nendawo”—neutral; “angivumelani”—I disagree) and Figure 6.

#### Evaluation 1

The main objective of the first evaluation was to refine the user requirements. The key objectives and results of this evaluation are shown in Textbox 2.

In total, 12 men participated in this evaluation, which was conducted as outlined earlier. In addition to the questions related to the key objectives outlined in Textbox 2, we also asked participants what the main messages were and whether there were any statements that should be added (ie, other considerations in deciding whether to engage in HIV care) and whether there were other ways the design could be improved. Overall, participants found it difficult to use the drag-and-drop option and did not understand the seesaw metaphor. Clicking was intuitive, and most participants preferred the images and text and the 2 options (agree or disagree). There was a consensus that the key message of this section was going to the clinic to start ART.

**Key objectives and results of evaluation 1.**

To test which interaction style participants preferred and found easier to use between a drag-and-drop version (dragging each statement into a bucket labeled *agree* or *disagree*) or a Likert scale version (where people clicked in a box labeled *agree*, *neutral* or *disagree*”)

- Participants found it difficult to use the drag-and-drop version, particularly more aged participants and participants with lower digital literacy levels
- A majority of participants needed to be shown how to drag-and-drop, despite the illustration at the beginning of the statements
- In contrast, clicking was intuitive; almost all the participants started clicking when they took the tablet

To determine whether the *neutral* option was valued by participants

- Some participants preferred drag-and-drop because it had 2 options (agree or disagree): “drag—I found it easy with the scale I was not completely sure of what was happening. With the drag option there was only two options which made it easy to select” [P6]

- Another participant reported that he found the neutral option confusing

To test whether participants preferred each option presented as text only or as text with an illustrative picture

- Most participants preferred text with an illustrative image to help them select the statements over the text only with many participants stating that images made it easier to understand the statements: “the image makes it clear what the statement says” [P7]
- Some found the images to facilitate engagement: “the images capture your attention” [P6]

To test whether participants were able to correctly interpret a seesaw metaphor, in which each response was stacked up on the end of a seesaw depending on whether it weighed toward or against attending a clinic (correct interpretation of the metaphor was that the end that was more heavily weighted and hence *down* was the preferred outcome)

- Participants did not understand the seesaw metaphor
- Many expressed confusion on how to interpret the results: “not sure how it should look like on the seesaw. When is it good, when it is up or down?” [P1]

To assess whether it was clear how to agree or disagree with a statement and whether it was clear how their selections related to the outcome of the seesaw

- It was not clear for some participants on how to agree or disagree with the statements; 2 participants (2/8) in the Likert scale option selected agree for all the statements

**Figure 7:**
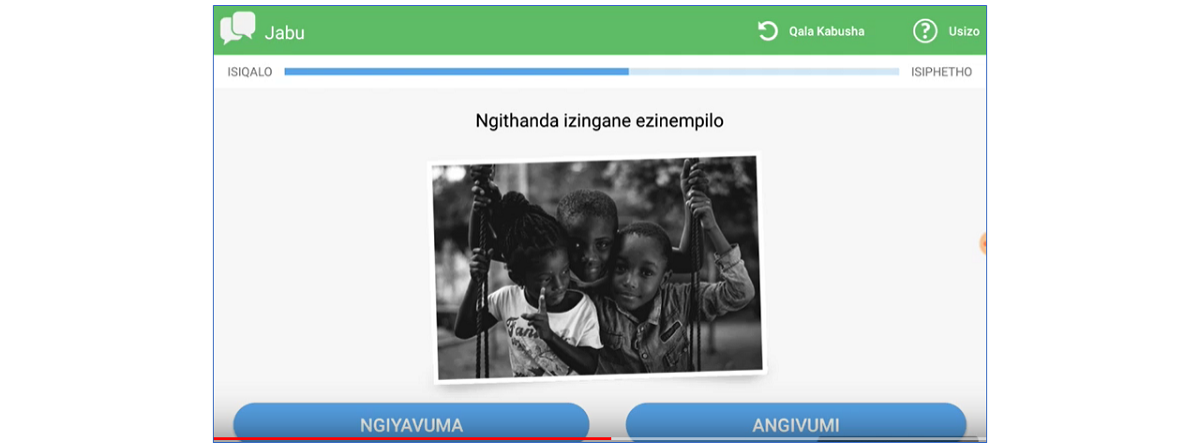
Example screenshot showing the design change with the 2 options, agree or disagree.

**Figure 8:**
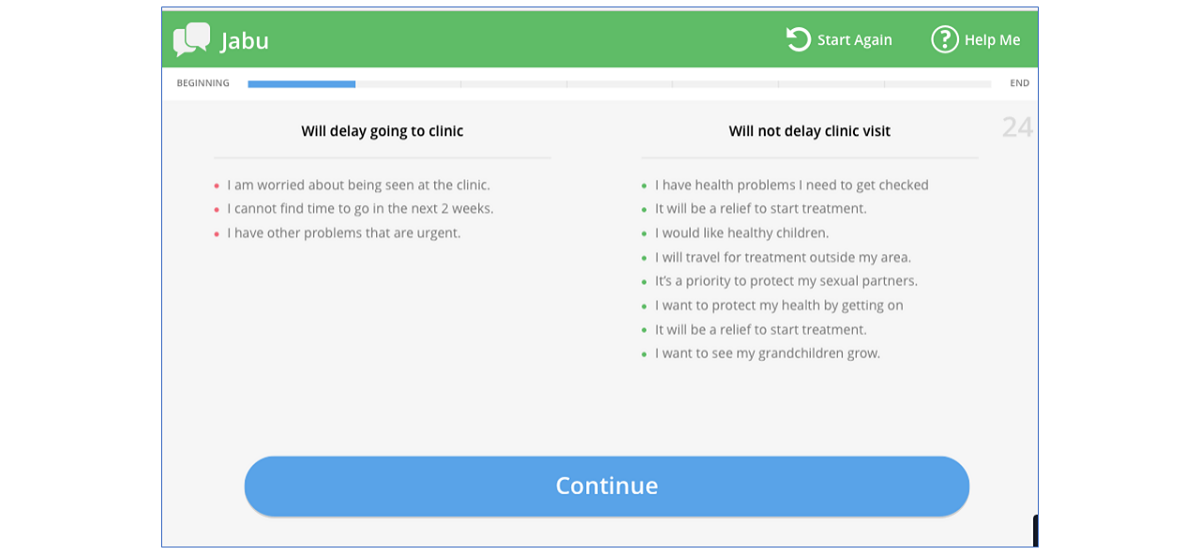
Example screenshot showing statements only without the seesaw and a clear label showing which side supports going to the clinic and which supports delaying (in English, but in isiZulu in the final app). This is a preliminary version, and the data were not final.

On the basis of the findings, the following design changes and decisions were made (as illustrated in Figures 7 and 8):

1. As the app was intended for one-off use, after seeing that clicking was intuitive and users struggled with the drag-and-drop, we decided to use clicking to select statements.
2. We limited the options to agree and disagree, as some participants did not find value in the neutral option.
3. To support users to make informed choices regarding attending a clinic for care, we used text and image as users expressed that images made it easier to understand the statements.
4. It was judged that the seesaw metaphor was not applicable for this exercise, as many participants struggled to understand what it meant if one side was more heavily weighted than the other. We removed the seesaw, summarized the statements, and explained what it means if they were skewed on one side. Furthermore, we labeled the right side as supporting going to the clinic in 2 weeks and left as delaying going to the clinic.
5. No further changes were judged necessary to address this question.

#### Evaluation 2

The main objective of the second evaluation is to validate the design changes made, particularly the summary screen shown in Figure 7. A total of 7 men participated in this evaluation cycle. We stopped them at the end of each section to ask guided questions: (1) Does the text only make the choices you selected clearer or do you think text and image would make it clearer? (2) What do you think is the key message of this section? (3) Would you prefer the agree and disagree buttons to be color coded?

##### Results of Evaluation 2

The design of the selection tool was received positively by all participants. All participants were selective on the statements they agreed or disagreed with as per their individual experiences, and they were able to interpret what it means if statements were weighted on one side (Figure 8). However, 2 participants mentioned that the statements were not clear or applicable to them. This could have been because the user was not affected by or living with HIV (the researcher did not ask the participant’s HIV status).

Of 7 participants, 4 preferred the text only in the summary page, validating the first evaluation findings that images were only important at the selection point. In addition, 6 participants reiterated that the key message of this section was about the importance of going to the clinic to start ART, with 1 participant adding that one should go to the clinic early and not wait until they are sick. Moreover, 5 participants said they would prefer the agree or disagree buttons to be color coded, with 4 participants suggesting that agree should be green and disagree, red:

colours help, maybe green and red so that they can see that they have made the right or wrong choice.

Participant 2

One participant stated that different colors would be particularly useful for people who cannot read.

**Figure 9:**
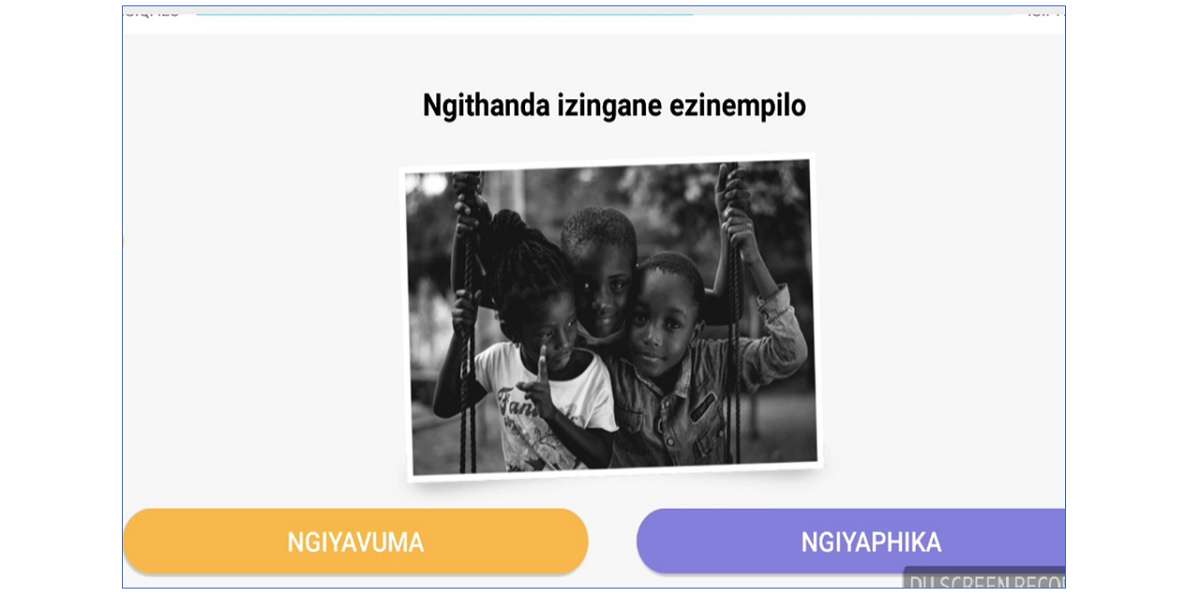
Design changes showing the neutral colors for agree and disagree.

On the basis of the preference for color coding and the association of green with right and red with wrong, we decided to use neutral colors that are not associated with right or wrong to help users make an individual choice as opposed to fearing being right or wrong (Figure 9).

#### Evaluation 3

This evaluation was conducted as part of the full Jabu section testing, mainly to assess understanding of the content as well as to check whether users find the presentation of content interesting and to validate the changes made as a result of evaluation 2. A total of 8 men participated in this cycle.

This evaluation did not reveal any usability issues, indicating that the changes made to the designs were effective. Participants were paying more attention before deciding whether they agreed or disagreed, and they listened to the audio while looking at the screen. Overall, participants reported finding the decision support tool engaging and useful. For example, when asked which part of the app they thought would be most relevant to their friends or other men, 3 participants referred to the clinic decision support tool. In addition, 1 participant, when asked if he learned anything new as a result of using the app, mentioned that he learned how to use a tablet, suggesting that there was incidental value (not directly related to the primary aim) in introducing such an app to people.

### Phase 3: Acceptability Testing (October 2018 to March 2019)

#### User Satisfaction Surveys

A total of 15 participants received EPIC-HIV 2 between October 2018 and January 2019, and 14 completed the user satisfaction surveys. Their mean age was 35.6 years (SD 13.38299), and a majority (8/14, 57%) had high school education, and half of them were unemployed. User satisfaction ratings were high (Table 1). Participants reported that they were generally satisfied with the app and found it to meet their needs for information on HIV treatment. All participants said that they received the kind of HIV management information that they wanted or expected and that they would definitely consider the app when seeking information about HIV management as well as recommending the app to friends in need of similar help. In addition, 86% (12/14) of participants were very satisfied with the amount of information that they received from the app. Overall, 79% (11/14) of the participants were very satisfied with the app by rating the simplicity and user friendliness of the app as excellent. The app has proven to empower the participants in managing their health; 86% (12/14) of participants definitely agreed.

#### In-Depth Interviews

A total of 14 men (aged 35-53 years) who had tested positive for HIV and had not linked to care within a month of receiving their test results received EPIC-HIV 2 between April 2018 and January 2019 agreed to be interviewed; 11 of them had linked to HIV care at the time of the interview. A majority of participants reported that the app offered relevant information about HIV management, boosted their confidence, and encouraged them to start and stay on treatment. However, this was not enough for all participants to link to HIV care. We report the experiences of men who used EPIC-HIV 2 using the SDT framework: autonomy, competence, and relatedness.

#### Complexities of the HIV Care Pathway: Examining Men’s Autonomy to Link to HIV Treatment

Participants described the app as motivating them to start ART by providing them with information on how to manage HIV to live a healthy life:

Mmm... I can say I have listened to the App from the beginning up to the end. Yes, I have really seen a need...I have seen it as a very important thing. I think that has encouraged me because I have never been to the clinic previously, but I went to clinic after that.

Participant 2, linked to care

For some, this was a catalyst to link to care (even if they did not act on it immediately):

I was supposed to visit the clinic a long time ago, but I didn’t because of work...I was thinking about that when I listened to EPIC [app]...you must be open as the EPIC was saying that you mustn’t fear that people will laugh at you. I took a decision because...this is life and we are living within a community...I realised that in order to be assisted, I must go to AHRI nurses at the clinic.

Participant 5, not linked to care

However, for some participants, the app alone was insufficient to motivate them to go to the clinic for HIV treatment. Other factors reduced the logistic barriers to linkage that nudged them to link. For some participants, it was the availability of our research nurses at local clinics, with the:

referral letter... that was going to make things easy for me...

Participant 4 linked to care

For others, such as participant 1, it was experiences such as the death of his best friend that motivated him to link to care after using the app:

Er...I can just say that there is nothing new I have learnt (from EPIC)...I started treatment because I've lived with people who had been using it (ARVs) before I knew my health status. One of my best friends passed away after he defaulted from taking treatment. After I found out that I am HIV positive I was motivated, and I said I will try by all means to take treatment accordingly. So, I can say I have learnt a lot from him about the risk of delaying...

Participant 1, linked to care

This suggests that for men to exercise autonomy to link to care, in addition to the app, external factors such as clinic operation hours, support, and individual experiences of HIV need to be considered.

### “I Am Not Giving Up on Life”: Men’s Competency and Decision to Manage HIV

Several of the participants reflected on how the positive narratives in the app encouraged them to take charge of their lives and manage HIV to improve their health outcomes and “avoid infecting other people with diseases” by describing “how to take the pills on time” and “comply with treatment.”

Some participants also reported that app messaging lessened their fears (particularly fear of disclosure) and motivated them to adopt positive attitudes to advance their health. The quotation below illustrates how some participants felt about their fears:

...I had that feeling of fear to say, oh my Lord how could I do this thing since I am afraid as it will be revealed? Eh, I also gained advices because they start with allowing you to listen from the messages that say once you know your status, you can get help so it’s up to you whether you do it or not. But I chose to be assisted because I am not giving up on life.

Participant 4, linked to care

Others also drew attention to the message of EPIC-HIV 2 that it gave them tips on how to discuss disclosure with their sexual partners:

Er...like disclosing your health status to your partner so that she can check for HIV as well for both of you to take treatment, yes, I think it’s good not to hide from your partner:

Participant 2, linked to care

Overall, for many participants, the app supported their competence to link to care through the positive messaging that gave hope, alleviated their fears, and reinforced the need for them to manage their health effectively to live a healthy life while minimizing the risk of transmitting HIV.

### Relatedness to Characters in the App: Men’s Perceptions of HIV Treatment and Management

Participants reflected how the narratives of different characters in the app reverberated with their present realities and how the app stories shaped their understanding of the importance of HIV treatment and management for them to live a healthy life:

...They [app characters] have mentioned the fact that once you have defaulted from taking treatment you will be very sick…the characters I have seen on the app were fit. They were sharing their experiences as to how they are taking their ARVs...

Participant 6, linked to care

They [app characters] were sharing simple information, something that is said even on TV by Ministers that you need not to be bedridden until you go to the clinic.

Participant 3, linked to care

Furthermore, a participant who had previously defaulted treatment had this to say:

The part I have seen as most essential one for me it’s where the characters were talking about the risk of defaulting from taking treatment...It motivated me to decide to be re-initiated on treatment...It is really necessary...because characters on the Epic were talking about things they had experienced.

Participant 2, linked to care

In sum, the empathetic nature of the personal testimonies of various characters in the app resonated with participants, and they felt cared for. Some of them described the relevance of the information shared through the app and were able to initiate treatment and took the decision to manage HIV to improve their health outcomes.

## Discussion

### Principal Findings

We found that it was possible to successfully apply a multiphased iterative development process to create a theoretically informed, interactive, tablet-based app to support men in making informed decisions about engaging with HIV care in a low-income, rural South African setting. Men in our setting who had been missing from the HIV care cascade found the app to be acceptable and reported that the stories in the app resonated with their realities and encouraged them to link to care. Previous work has established that digital interventions are acceptable and effective in improving clinic attendance, ART adherence, and turnaround time from testing to treatment [11,14,15]. Our results underscore the value of using a person-based approach to integrate evidence-based content and design to ensure that the app is relatable and addresses the local perception of HIV care. Using HCI techniques ensured that the app was simple, usable, and engaging for end users with varying levels of education, health, and digital literacy. For example, user testing established that the *seesaw* metaphor was difficult for participants to understand, so it was removed between the first and the second iterations of app testing. In addition, supporting different learning styles and motivations through tailoring of content provided a greater level of engagement for participants. Previous studies have shown that interactive digital interventions that deliver tailored content that address the specific challenges for individual users can be highly engaging and likely to be understood [51]. Furthermore, using digital technologies could help people learn new skills (using a tablet) and facilitate a sense of accomplishment that can improve their competence and possibly improve engagement.

Drawing on behavior change theory, such as SDT, ensured that EPIC-HIV 2 supported individual decision making. There is an increasing need to apply evidence-based theories to enhance the development of digital interventions and improve their efficacy [15,25,52]. Our findings corroborate previous studies that have used the SDT framework (autonomy, competence, and relatedness) to understand ART adherence and treatment motivation and overall health behavior among men living with HIV [23,24]. However, the app was insufficient as a *stand-alone intervention* for men in our sample to exercise their full autonomy to link to HIV care without other factors such as it being convenient to initiate treatment, individual experiences of HIV, and support. Combining tailored digital interventions with other interventions to address a range of barriers to HIV care, especially supply-side barriers, should be considered in the future to close the present *linkage gap* in the HIV treatment cascade.

### Study Strengths and Limitations

Using a mixed methods approach to understand usability and acceptability and the potential of the app to encourage men to link to care are strengths of the study. However, the generalizability of study findings outside our area may be limited because of the locally tailored design of the EPIC-HIV 2 app, the sampling method, sample size, and specific study sites. In addition, the possibility of social desirability bias cannot be excluded as some participants might have found it easier to say positive things about the app and not necessarily translate to the actual initiation of ART. Furthermore, we cannot establish what the outcome would have been if we had made different design decisions or applied a different theory of behavior change. The decision to develop a tablet-based app was fitting for the specific context of use, but more widespread use of such an app would probably be achieved by implementing it on mobile phones for independent use; this remains an area for future work.

### Conclusions

Using a multidisciplinary approach and drawing on evidence-based theories to develop digital interventions can ensure that the resultant products are acceptable and engage in a wide range of users. Our work aims to pave the way for a greater focus on mixed methods and person-centered approaches to the development of digital interventions for HIV care.

## Appendix

Multimedia Appendix 1 Literature review and secondary analysis.

Multimedia Appendix 2 User evaluation questions and guides.

Multimedia Appendix 3 Self-determination theory app content and design.

Multimedia Appendix 4 Example of navigation pathway in the Jabu module.

## Abbreviations

AHRI: Africa Health Research Institute
ART: antiretroviral therapy
ARV: antiretroviral
CAB: community advisory board
CSQ: Client Satisfaction Questionnaire
EPIC-HIV: Empowering people through informed choices for HIV
FGD: focus group discussion
HCI: human-computer interaction
HITS: home-based intervention to test and start
PE: public engagement
SDT: self-determination theory
