# Supplementary material for: Development and Acceptability of a Tablet-Based App to Support Men to Link to HIV Care: Mixed Methods Approach
Source: JMIR Mhealth Uhealth. 2020 Nov 24;8(11):e17549. doi: 10.2196/17549 (PMC7723744; doi:10.2196/17549)
Supplement: Multimedia Appendix 1 [file mhealth_v8i11e17549_app1.pdf]

Paper coding format:	Literature Synthesis: Qualitative Review	Literature synthesis: Quantitative Review	Literature synthesis: Mixed methods	Secondary analysis used in development of EPIC-HIV 2 (Uvo lwakho)	Existing Research Team Knowledge	Editorial Opinion Papers

Barrier to overcome	Key References	Key finding	Modifiable?
**Supply side barriers**			
Clinics 'feminised' spaces where men do not feel comfortable	Cox, V., Campbell, A., Raphahlelo, N., McIntyre, J., & Rebe, K. (2013).	77% of the respondents would attend male clinic for ART initiation within one week<br>99% preferred attending male only clinics for ART services, even if waiting time increased<br>49% avoided clinic attendance because of female staff	Not directly modifiable in EPIC-HIV2 except by supporting men to see the long term benefits as outweighing the social costs and offering ideas for strategies to overcome embarrassment in the clinic
	Groh, K., Audet, C. M., Baptista, A., Sidat, M., Vergara, A., Vermund, S. H., & Moon, T. D. (2011). Barriers to antiretroviral therapy adherence in rural Mozambique. BMC public health, 11(1), 650. DOI: 10.1186/1471-2458-11-650	Men expressed a greater concern about poor treatment by health care workers than women (83% men vs. 0% women).	
	Secondary analysis: Unpublished Uvo lwakho data	"Females go for testing if they are infected they go to the clinic and get treatment, but as males we don't like to visit the clinic" (22-year-old male unemployed)	
	Heestermans, T., Browne, J. L., Aitken, S. C., Vervoort, S. C., & Klipstein-Grobusch, K. (2016). Determinants of adherence to antiretroviral therapy among HIV-positive adults in sub-Saharan Africa: a systematic review. BMJ global health, 1(4), e000125. 10.1136/bmjgh-2016-000125	Dissatisfaction with healthcare facility and healthcare workers	
Lack of confidentiality if seen in clinic	Groh, K., Audet, C. M., Baptista, A., Sidat, M., Vergara, A., Vermund, S. H., & Moon, T. D. (2011). Barriers to antiretroviral therapy adherence in rural Mozambique. BMC public health, 11(1), 650.	Community participants in focus groups noted a lack of confidentiality and poor treatment by hospital staff (42%)	Not directly modifiable in EPIC-HIV2 except by supporting men to see the long term benefits as outweighing the social costs and

	DOI: 10.1186/1471-2458-11-650		offering ideas for strategies to increase confidentiality
	Secondary analysis: Unpublished Uvo lwakho data	"If you meet a person at clinic when they leave they will go around saying they saw me in that room maybe I was there for testing. They will say so and so is now eating it (taking treatment) has to be because she was dating so and so" (42-year-old male, PLHIv)	
	Secondary analysis: Unpublished Uvo lwakho data	"I don't go to the Clinic, I am first scared. The first thing you will do is to look at people you are close with…and this also makes people not to want to go to clinics. You see here, you see if you are going up the container (park home), you just see that… You feel crazy, and scared to check your status. That happens to many men"" (46-year-old male, self-employed)	
	Fitzgerald, M., Collumbien, M., & Hosegood, V. (2010). "No one can ask me 'Why do you take that stuff?'": men's experiences of antiretroviral treatment in South Africa. AIDS care, 22(3), 355-360. http://dx.doi.org/10.1080/09540120903111536	Participants in the study held strong views to appropriate and professional behaviour of staff regarding confidentiality	
Long waits at busy clinics put men off going	Secondary analysis: Unpublished Uvo lwakho data	"they use traditional medicine because they know that if they go to the clinic they will not get help fast" (35-year-old male, unemployed)	Not directly modifiable in EPIC-HIV2 except by supporting men to see the long term benefits as outweighing the social costs and offering ideas for strategies to reduce wait
Clinics open at times men find it difficult to go there	Cornell, M., Cox, V., & Wilkinson, L. (2015). Public health blindness towards men in HIV programmes in Africa. Tropical Medicine & International Health, 20(12), 1634-1635. DOI: 10.1111/tmi.12593	among 200 male clients, 99% would prefer attending a male clinic for ART services and 96% requested extended clinic hours and close proximity to home or public transport hubs. Informed by this survey, a once weekly male after-hours service was started in an existing large ART clinic in Khayelitsha	Not directly modifiable in EPIC-HIV2 except by supporting men to see the long term benefits as outweighing the social costs and offering ideas for strategies to attend clinics at different times or use different clinics
Poor relationships with health care workers	Seconday analysis: Uvo lwakho data Unpublished	They (nurses) shout at them (patients) and talk anyhow like when diabetic elderly people pee on themselves" (21-year old male, unemployed)	Not directly modifiable in EPIC-HIV2 except by supporting men to see the long term benefits as

	Groh, K., Audet, C. M., Baptista, A., Sidat, M., Vergara, A., Vermund, S. H., & Moon, T. D. (2011). Barriers to antiretroviral therapy adherence in rural Mozambique. BMC public health, 11(1), 650. DOI: 10.1186/1471-2458-11-650	Men expressed a greater concern about poor treatment by HCW than women (83% men vs. 0% women).	outweighing the social costs, and offering ideas for strategies to manage less respectful treatment by health care workers
	Bhagwanjee, A., Govender, K., Akintola, O., Petersen, I., George, G., Johnstone, L., & Naidoo, K. (2011). Patterns of disclosure and antiretroviral treatment adherence in a South African mining workplace programme and implications for HIV prevention. African Journal of AIDS Research, 10(sup1), 357-368. http://dx.doi.org/10.2989/16085906.2011.637737	In this study, participants reported that health care providers played a significant role in allaying fears and reinforcing treatment-adherence behaviour	
	Secondary analysis: Unpublished Uvo lwakho data	“No…I would say they are not enough because even at the clinics the healthcare professionals do not advice the patients on how and when to take their treatment and the consequences of not taking it. People just go there to collect their treatment because they have run of it” (20-year-old female, unemployed)	
**Demand-side barriers**			
Concern that if HIV+ will be shunned, presence of stigma whereby HIV not openly discussed	Bhagwanjee, A., Govender, K., Akintola, O., Petersen, I., George, G., Johnstone, L., & Naidoo, K. (2011). Patterns of disclosure and antiretroviral treatment adherence in a South African mining workplace programme and implications for HIV prevention. African Journal of AIDS Research, 10(sup1), 357-368. http://dx.doi.org/10.2989/16085906.2011.637737	Over one-third (of a sample of 19) of the participants asserted the need to ‘be strong’ for their family in order to protect them	Offer strategies to overcome stigma through openness and acceptance
Fear of death and illness	Cornell, M., Cox, V., & Wilkinson, L. (2015). Public health blindness towards men in HIV programmes in Africa. Tropical Medicine & International Health, 20(12), 1634-1635. DOI: 10.1111/tmi.12593	Simply being in care appeared to be protective for men, possibly through access to other preventive and curative services that could reduce non-HIV mortality.	Offer narratives from ‘men they may identify with’ of being well and able to live a valued life in the way one wants because of taking ART long-term

Concern that if HIV+ will be ‘blamed’ for sexual ‘misconduct’ and isolated from family	Mills, E. J., Beyrer, C., Birungi, J., & Dybul, M. R. (2012). Engaging men in prevention and care for HIV/AIDS in Africa. PLoS medicine, 9(2), e1001167. 10.1371/journal.pmed.1001167	Evidence indicating that men may feel that they have been caught at their hidden sexual behaviours and so they avoid HIV testing	Offer narratives from ‘men they may identify with’ of how they explained their status to family members and continued to manage relationships.
Feeling healthy (so not understanding the need for a test or linkage to care),	Kumwenda, M., Munthali, A., Choko, A., Chikovore, J., Nliwasa, M., Sambakunsi, R., … & Corbett, E. L. (2016). The influence of masculinity on HIVST community intervention: a qualitative evaluation of empirical evidence from Blantyre, Malawi. www.hsrc.ac.za/en/research-data/view/8193	Perception of good health making men not want to test/know their HIV-status, even those who know their partner’s HIV positive status.	Offer narratives from ‘men they may identify with’ of how they despite feeling well they tested and then linked to care and are pleased they did.
A preference for traditional medicine	Chikovore, J., Gillespie, N., McGrath, N., Orne-Gliemann, J., Zuma, T., & ANRS 12249 TasP Study Group. (2016). Men, masculinity, and engagement with treatment as prevention in KwaZulu-Natal, South Africa. AIDS care, 28(sup3), 74-82. http://dx.doi.org/10.1080/09540121.2016.1178953	Observations that men preferred traditional medicine and that primary health centres were not welcoming to men, descriptions that men used lay measures to ascertain HIV status.	Offer narratives from ‘men they may identify with’ who used traditional medicine but also then used ART to better manage HIV.
	Heestermans, T., Browne, J. L., Aitken, S. C., Vervoort, S. C., & Klipstein-Grobusch, K. (2016). Determinants of adherence to antiretroviral therapy among HIV-positive adults in sub-Saharan Africa: a systematic review. BMJ global health, 1(4), e000125. 10.1136/bmjgh-2016-000125	Use of traditional/herbal medicines	
The need to maintain ‘reputational’ identity, at least with other men, as strong, with capacity for hard work and earning potential, and sexual prowess	Chikovore, J., Gillespie, N., McGrath, N., Orne-Gliemann, J., Zuma, T., & ANRS 12249 TasP Study Group. (2016). Men, masculinity, and engagement with treatment as prevention in KwaZulu-Natal, South Africa. AIDS care, 28(sup3), 74-82. http://dx.doi.org/10.1080/09540121.2016.1178953	The accounts detailed men’s unwillingness to engage with HIV testing and care, seemingly tied to their pursuit of valued masculinity constructs such as having strength and control, being sexually competent, and earning income.	Offer narratives from ‘men they may identify with’ who maintained identities as respect as sportsman, hard worker and supporter of the family, of community leader, at the same time as being HIV+ and taking ART.
	Marson, K. G., Tapia, K., Kohler, P., McGrath, C. J., John-Stewart, G. C., Richardson, B. A., … & Chung, M. H. (2013). Male, mobile, and moneyed: loss to follow-up vs. transfer of care in an urban African	The most common reason for loss to follow-up was moving residence, predominantly due to job loss or change in employment.	

	antiretroviral treatment clinic. PLoS One, 8(10), e78900. https://doi.org/10.1371/journal.pone.0078900		
The ability to maintain 'respectable' identity as healthy and able to provide for a family.	Chikovore, J., Hart, G., Kumwenda, M., Chipungu, G. A., & Corbett, L. (2015). 'For a mere cough, men must just chew Conjex, gain strength, and continue working': the provider construction and tuberculosis care-seeking implications in Blantyre, Malawi. Global health action, 8(1), 26292. http://dx.doi.org/10.3402/gha.v8.26292	Role descriptions by both men and women in the study universally assigned men as primary material providers for their immediate family, that is, the ones earning and bringing livelihood and additional material needs. In a context where collectivism was valued, men were also expected to lead the provision of support to wider kin. Successful role enactment was considered key to achieving recognition as an adequate man; at the same time, job scarcity and insecurity, and low earnings gravely impeded men.	Offer narratives from 'men they may identify with' who maintained identities as respect as sportsman, hard worker and supporter of the family, of community leader, at the same time as being HIV+ and taking ART.
	Hosegood, V., Richter, L., & Clarke, L. (2016). "... I Should Maintain a Healthy Life Now and Not Just Live as I Please..." Men's Health and Fatherhood in Rural South Africa. American journal of men's health, 10(6), NP39-NP50. DOI: 10.1177/1557988315586440	The study reveals the pervasiveness of an ideal of healthy fathers/men, one in which the health of men has practical and symbolic importance not only for men themselves but also for others in the family and community.	
	Fitzgerald, M., Collumbien, M., & Hosegood, V. (2010). "No one can ask me 'Why do you take that stuff?'": men's experiences of antiretroviral treatment in South Africa. AIDS care, 22(3), 355-360. http://dx.doi.org/10.1080/09540120903111536	Study reported concerns from men about being unable to meet strongly gendered expectations in relation to family and work	
	Siu, G. E., Seeley, J., & Wight, D. (2014). 'Dented'and'Resuscitated'masculinities: the impact of HIV diagnosis and/or enrolment on antiretroviral treatment on masculine identities in rural eastern Uganda. SAHARA: Journal of Social Aspects of HIV/AIDS Research Alliance, 11(1), 211-221. DOI:10.1080/17290376.2014.986516	HIV infection and illness dented masculinity as men lost authority within the domestic sphere. A weakened provider role and over reliance on wives and children undermined masculinity as family head, and social sanctioning of their sexual activity, undermined conventional masculine identities predicted on reputation.	
	Mills, E. J., Beyrer, C., Birungi, J., & Dybul, M. R. (2012). Engaging men in prevention and care for HIV/AIDS in Africa. PLoS medicine, 9(2), e1001167. 10.1371/journal.pmed.1001167	If the head male member contracts HIV and does not receive the appropriate care, ill health or death of this individual can severely impact household family income.	

Homophobia	Mills, E. J., Beyrer, C., Birungi, J., & Dybul, M. R. (2012). Engaging men in prevention and care for HIV/AIDS in Africa. PLoS medicine, 9(2), e1001167. 10.1371/journal.pmed.1001167	The reality that men are less likely to seek health care is intimately linked to perceptions of masculinity, and is generally considered to be part of the same phenomenon that drives multiple partnering, violence against women, substance use, and homophobia among men	Not dealt with in EPIC-HIV2
Belief that medicine should be curative. Concept of lifelong chronic illness relatively a new idea.....use of ART even if asymptomatic	Bhagwanjee, A., Govender, K., Akintola, O., Petersen, I., George, G., Johnstone, L., & Naidoo, K. (2011). Patterns of disclosure and antiretroviral treatment adherence in a South African mining workplace programme and implications for HIV prevention. African Journal of AIDS Research, 10(sup1), 357-368. http://dx.doi.org/10.2989/16085906.2011.637737	Feeling better on ARV treatment resulted in some individuals stopping their ARV medication for a period of time	Offer narratives from 'men they may identify with' who maintained identities as respect have come to understand HIV as a chronic illness to be treated long-term.
Losing control/feeling compromised	Bhagwanjee, A., Govender, K., Akintola, O., Petersen, I., George, G., Johnstone, L., & Naidoo, K. (2011). Patterns of disclosure and antiretroviral treatment adherence in a South African mining workplace programme and implications for HIV prevention. African Journal of AIDS Research, 10(sup1), 357-368. http://dx.doi.org/10.2989/16085906.2011.637737	The non-adherent sample (n = 15 out of 19) clearly struggled to come to terms with their HIV infection and expressed being less in control of their lives.	Offer narratives from 'men they may identify with' who managed to maintain control because they took ART long-term.
	Boullé, C., Kouanfack, C., Laborde-Balen, G., Boyer, S., Aghokeng, A. F., Carrieri, M. P., … & Peytavin, G. (2015). Gender differences in adherence and response to antiretroviral treatment in the Stratall Trial in rural district hospitals in Cameroon. JAIDS Journal of Acquired Immune Deficiency Syndromes, 69(3), 355-364. doi: 10.1097/QAI.0000000000000604	Study provides important evidence that African men are more vulnerable to ART failure than women and that the male vulnerability extends beyond adherence issues.	
Alcohol use	Fitzgerald, M., Collumbien, M., & Hosegood, V. (2010). "No one can ask me 'Why do you take that stuff?'": men's experiences of antiretroviral treatment in South Africa. AIDS care, 22(3), 355-360. http://dx.doi.org/10.1080/09540120903111536	Alcohol use and abuse by men themselves or by family members was found to be an important issue influencing disclosure, uptake and adherence. Men reported self-imposed delays to enrolment while they tried to stop or reduce alcohol use, although none had sought advice or professional help in doing so	Offer narratives from 'men they may identify with' to make it clear that some beers are fine but what to do if forget to take ART one day. But EPIC-HIV2 does not

			directly deal with problem of alcohol mis-use
		Employment-related migration will keep men away from their partner and families for long time periods, and this absence may make them more vulnerable to HIV infection due to sexual exposure, drug and alcohol use, and delinkages with local health services	
	Heestermans, T., Browne, J. L., Aitken, S. C., Vervoort, S. C., & Klipstein-Grobusch, K. (2016). Determinants of adherence to antiretroviral therapy among HIV-positive adults in sub-Saharan Africa: a systematic review. BMJ global health, 1(4), e000125. 10.1136/bmjgh-2016-000125	Main determinants of non-adherence were use of alcohol, male gender	
