# Supplementary material for: Development and Acceptability of a Tablet-Based App to Support Men to Link to HIV Care: Mixed Methods Approach
Source: JMIR Mhealth Uhealth. 2020 Nov 24;8(11):e17549. doi: 10.2196/17549 (PMC7723744; doi:10.2196/17549)
Supplement: Multimedia Appendix 2 [file mhealth_v8i11e17549_app2.pdf]

EPIC 2: Seesaw Evaluation

Greetings

We are part of a study called HITS (Home-based Intervention to Test and Start), conducted by Africa Health Research Institute (formerly known as Africa Centre). The HITS study is interested in finding ways to reduce the spread of HIV infection and reduce the number of unnecessary deaths due to HIV. In an attempt to do this, one of the other things that HITS will do, will offer an HIV decision support application in a tablet to encourage men to test and link for HIV care. But this is the formative phase. We want to get more information to improve the app.

You are invited to participate because your community is part of the communities randomly allocated to participate in the study

Participating in the study is completely voluntary and you can withdraw at any time without negative consequences

What do we want to learn today?

This is a new app that fieldworkers will take to the households in the community. It is for men and is about HIV. We are going to show you one section of the app that needs to be improved. We would like to get your opinion to see how it can be made better. To do this, we will ask you to use the app and afterwards we will ask you questions about it. The app is not finished, so you can help us by telling us what you like and what you don't like about our ideas. If you have other suggestions, please let us know those.

We are especially interested in what you think about the design and how we can make it better. We will ask you questions about the design options, but please remember there are no right or wrong answers and there is no right or wrong way to use the app. This app is not perfect. We are doing this to improve it, so anything you tell us is very valuable and can help us make it better. We would really appreciate your honest feedback

There will be 2 tasks and the entire process should take around 15 minutes. You can stop at any time and ask questions if anything is unclear

Consent

Can we confirm that you are happy for this to be recorded, all information will be kept confidential?

Date	
Participant number	
Consent for recording and participation	
Group	**GROUP 1:** Design 1, then Design 2<br><br>**GROUP 2:** Design 2, then Design 1

Turn on screen and voice recorder

Task 1

Please start using the app, I will tell you where to stop.

[Show first design option and then ask questions at the end.]

Design option 1 / Design option 2	
Take notes on interaction	
Is it clear how to select or choose a statement that is your priority? Please could you describe it.	
Was it clear how the priorities you selected related to the outcome of the seesaw? Please could you describe it.	
What do you think the key message of the app was?	
What aspect of the app do you think would be most relevant to your friends or other men?	

Task 2

Thank you, now we will start task two. It is very similar to task one. Please start using the app

[Then show second design and ask question at the end]

Design option 1 / Design option 2	
Take notes on interaction	
Which version did you prefer? Why?	
Which version made selecting the priorities more clear? Why?	
Which version made the results more clear? Why?	

Thank you, this is really helpful. Now we will continue.

Just a few questions to finish off.

Can you think of other ways the design can be improved?	
Do you have any other comments?	

Thank you very much for participating, it is really helpful. We'll be able to show you the app when we have made improvements
