# Supplementary material for: Development and Acceptability of a Tablet-Based App to Support Men to Link to HIV Care: Mixed Methods Approach
Source: JMIR Mhealth Uhealth. 2020 Nov 24;8(11):e17549. doi: 10.2196/17549 (PMC7723744; doi:10.2196/17549)
Supplement: Multimedia Appendix 3 [file mhealth_v8i11e17549_app3.pdf]

EPIC-HIV 2 components	Design objectves	SDT relevance
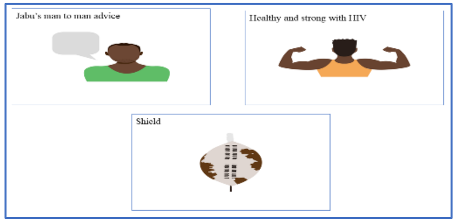	Offers a choice to 1 or all three modules : 1) Jabu'man to man advice; 2) healthy and strong with HIV, and 3) the shield	Offering choice to support autonomy
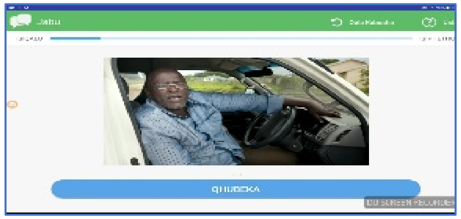	Personal testimonies from local men living with HIV: Jabu – "*My name is Jabu. I think of the virus like a visitor I don't want. HIV is never going to to leave my body, but I can control it. With understanding comes control*"	Supporting relatedness by using local men from the community that the user can identify with. This can further bolster social support (feeling of being cared for)
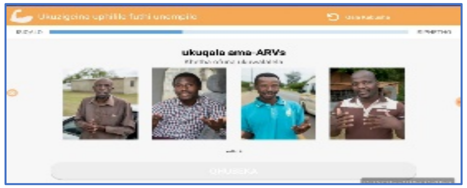	Heathy and strong –"*You wish to keep your health and strength. You have taken the first step by knowing your status What do you need to do next?*" Then 4 men give their personal stories on 4 topics: 1) how to get to the clinic and finding your way around the clinic; 2) disclosure; 3) starting ARVs, and 4) staying on ARVs	
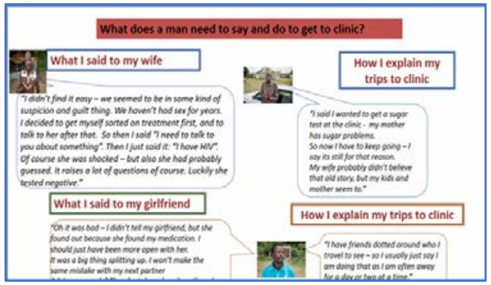	Offer choice to listen to 1 or all 4 men. Different men giving their stories on how they started ART: HS1 - "*For the first few months I used my phone as a reminder to take my daily medication. I try to keep a couple of spare pills wrapped up in my wallet in case I forget to take them away with me*" HS2 – "*My first nurse was so abrupt – rude really. She seemed to be being judgmental. So I decided it was all*	Supporting autonomy by offering choice (option to listen to 1 or all 4 men). Also, supports competence through providing examples of men taking control and providing guidance on how they started ART, deal with disclosure, continue to take ART.

	the more important to keep going – I mean why become ill just because of a person like that? We are used to each other now. The nurses have a tough job for sure”<br>HS3- *“I struggled at first – I had left things way too late and got TB. For me that treatment ended up heavy duty, as it was resistant to many medications and I had to take many pills a day. Its much easier now I am on one pill a day”*<br>HS4 – *“I had some side effects with ARVs at first – slight nausea and slight diarrhoea – but that all settled after a couple of weeks. So hang on in there.”*	
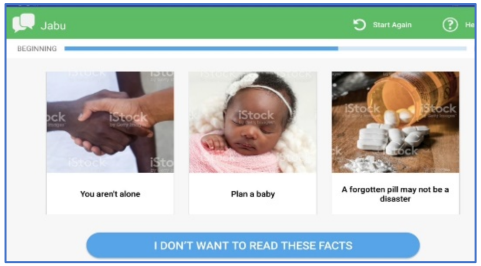	Jabu gives his man to man advice on how he views HIV as an uninvited visitor and how he controls it with ART.<br>*“I think of HIV as a visitor I don’t want. A visitor who might harm me. A visitor that will never leave.<br>But now HIV must submit to my control, because I am using daily ARVs against it. With my viral load suppressed I will stay strong. With my viral load suppressed I will not pass the virus on. No one sees me as a pushover – that virus included!”*	Supporting competence by allowing me to see a positive future with HIV - promoting motivation for desired behaviours. Also, provides tips on how to live positively with HIV
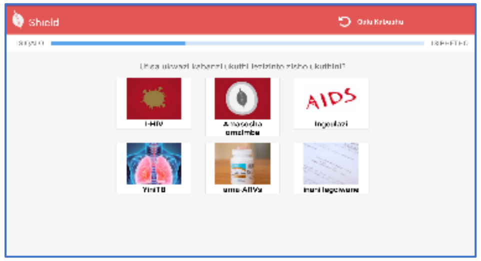	The shield sexplain the basic concepts and terminology used in HIV management (the shield uses graphic designs to describe how ART fights HIV in the body using 6 common terms used in HIV management).<br>*‘ARVs – antivirus medication – is the name of the pills that kill HIV. It can also be called ART, it’s the same thing. ARVs are forever – but usually, its just one pill a day. And these days most people have no unwanted*	Supporting competence by using graphic design to explain certain HIV and ART concepts

	effects from the medication If they do they are mild (like bad dreams), and usually side effects stop in the first 4 weeks. Its true that some of the older treatments did cause problems more commonly, but things are improving all the time. Taking ARVs regularly means you can have great control over the virus, and your soldier cells (CD4 cells) get a chance to recover, and their numbers will rise However if you start ARVs at a very late stage, it can be very difficult for your soldier cells to start to multiply again. This is why it is best not to delay. ARVs are now offered to everyone with HIV, however many soldier cells they have, because it is proven that it is good for your health to start treatment early. The important sign that ARVs are working effectively is that the amount of virus in your blood – the viral load – falls a great deal by 6 months of taking medication regularly. Tell me more about the viral load.'	
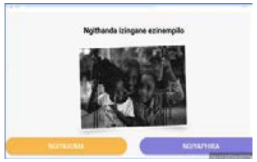 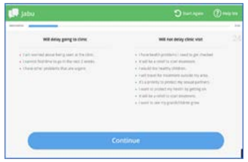	Decisional support tool to assess readiness to attend a clinic in two weeks. Interactive and choice to agree or disagree with the statements	Offering choice to support autonomy whilst at the same time supporting competence
