# Supplementary figures and images for: Development and Acceptability of a Tablet-Based App to Support Men to Link to HIV Care: Mixed Methods Approach
Source: JMIR Mhealth Uhealth. 2020 Nov 24;8(11):e17549. doi: 10.2196/17549 (PMC7723744; doi:10.2196/17549)

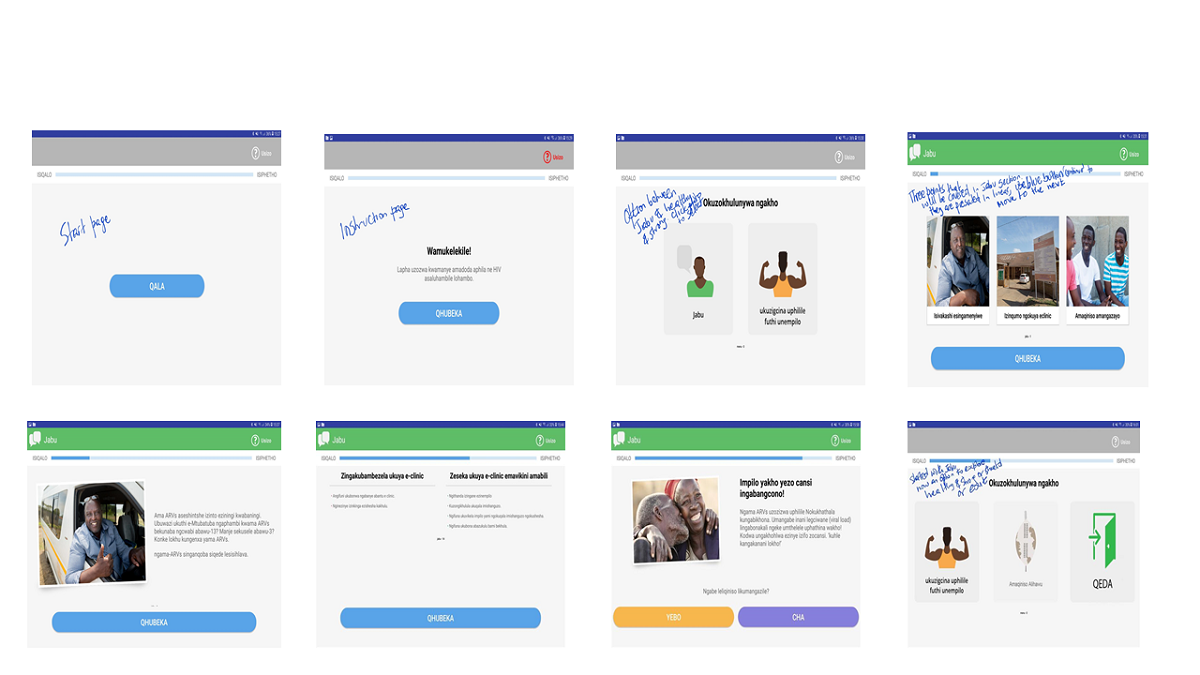

Supplement: Multimedia Appendix 4 [file mhealth_v8i11e17549_app4.png]
